# Coupling Mixed Mode Chromatography/ESI Negative MS Detection with Message-Passing Neural Network Modeling for Enhanced Metabolome Coverage and Structural Identification

**DOI:** 10.3390/metabo11110772

**Published:** 2021-11-11

**Authors:** Gang Xing, Vishnu Sresht, Zhongyuan Sun, Yuji Shi, Michelle F. Clasquin

**Affiliations:** 1 Internal Medicine Research Unit, Pfizer Worldwide Research, Development & Medical, Cambridge, MA 02139, USA; Zhongyuan.Sun@pfizer.com (Z.S.); Yuji.Shi@pfizer.com (Y.S.); 2 Simulation and Modeling Sciences, Pfizer Worldwide Research, Development & Medical, Cambridge, MA 02139, USA; Vishnu.Sresht@pfizer.com

**Keywords:** metabolomics, LCMS, retention time prediction, machine learning, MPNN

## Abstract

A key unmet need in metabolomics continues to be the specific, selective, accurate detection of traditionally difficult to retain molecules including simple sugars, sugar phosphates, carboxylic acids, and related amino acids. Designed to retain the metabolites of central carbon metabolism, this Mixed Mode (MM) chromatography applies varied pH, salt concentration and organic content to a positively charged quaternary amine polyvinyl alcohol stationary phase. This MM method is capable of separating glucose from fructose, and four hexose monophosphates a single chromatographic run. Coupled to a QExactive Orbitrap Mass Spectrometer with negative ESI, linearity, LLOD, %CV, and mass accuracy were assessed using 33 metabolite standards. The standards were linear on average >3 orders of magnitude (R^2^ > 0.98 for 30/33) with LLOD < 1 pmole (26/33), median CV of 12% over two weeks, and median mass accuracy of 0.49 ppm. To assess the breadth of metabolome coverage and better define the structural elements dictating elution, we injected 607 unique metabolites and determined that 398 are well retained. We then split the dataset of 398 documented RTs into training and test sets and trained a message-passing neural network (MPNN) to predict RT from a featurized heavy atom connectivity graph. Unlike traditional QSAR methods that utilize hand-crafted descriptors or pre-defined structural keys, the MPNN aggregates atomic features across the molecular graph and learns to identify molecular subgraphs that are correlated with variations in RTs. For sugars, sugar phosphates, carboxylic acids, and isomers, the model achieves a predictive RT error of <2 min on 91%, 50%, 77%, and 72% of held-out compounds from these subsets, with overall root mean square errors of 0.11, 0.34, 0.18, and 0.53 min, respectively. The model was then applied to rank order metabolite IDs for molecular features altered by GLS2 knockout in mouse primary hepatocytes.

## 1. Introduction

The study of metabolites, their perturbation in disease, and correction with therapeutics is central to our understanding of disease biology and pharmacology. In recent years, the field of metabolomics has been greatly enabled by technological advances in high-resolution accurate mass spectrometers. Although Mass Spectrometry provides compelling specificity in assignment of a chemical formula, continuing to advance separation chemistry is required for accurate metabolite identification and quantitation. Of particular challenge are the hydrophilic metabolites of central carbon metabolism, including simple sugars, sugar phosphates, carboxylic acids, and related amino acids, which are inherently incompatible with traditional Reverse Phase (RP) retention mechanisms.

To date, the LC retention of these hydrophilic and often charged metabolites has been achieved by normal phase (NP) [1], Ion Exchange (IE) [2], Hydrophilic Interaction Chromatography (HILIC) [3,4], and RP-ion pair (RP-IP) LC [5,6], where the latter two are more commonly coupled to MS detection due to solvent compatibility. A variation of normal phase chromatography, HILIC applies highly organic solvents with a small aqueous component to a polar stationary phase [7]. By increasing the aqueous content of the mobile phase, analytes are eluted in order of increasing polarity. This technique was extended to an ion-exchange column coupled to a highly organic mobile phase with Electrostatic Repulsion HILIC (ERLIC) [8]. By superimposing two independent retention mechanisms—hydrophilic interaction and electrostatic repulsion—ERLIC can be tuned for the retention of neutral, acidic and basic analytes on a charged stationary phase by manipulating mobile phase organic content, pH, and salt concentration, and to date has had success in (phospho)proteomics.

Although multiple HILIC, IEC, and RP-IP metabolomics methods have been described for the detection of sugar mono- and bis-phosphates of glycolysis and the pentose phosphate pathway [4,6,9,10,11], most fail to detect the essential nutrient input glucose, which commonly co-elutes with other hexose isomers in the solvent front. Successful retention of sugars and sugar phosphates has been described with pre-column derivatization [12], which cannot be universally applied to the rest of the central carbon metabolome. A significant advancement was recently published by Mathon et al., applying HILIC mobile phase with trimethylamine and methylphosphonic acid modifiers to a bridged-ethylene hybrid amide column to retain both sugars and sugar phosphates under alkaline conditions [13], albeit without separation of sugar isomers. As exemplified by Mathon et al., continually pushing the boundaries of traditional chromatographic techniques is required for improved metabolic coverage and isomer separation.

In addition to the challenges of separation chemistry, another major hurdle to metabolomics data interpretation is improving the confidence in identity assignment for MS features which match more than one metabolite in a database. While matching based on exact mass, isotope abundance pattern [14], and MS^2^ has been enabled by programs such as XCMS [15], mzMINE [16], SIEVE^TM^, CSI:FingerID [17], and others [18,19,20], each fails to apply knowledge of one’s own chromatographic retention mechanism. One creative solution to informing metabolite ID, bypassing MS^2^ and isotope abundance patterns, is by conducting an integrative network-based analysis based on exact mass alone and allowing resultant networks to inform the assignment of metabolites [21]. Approaches such as this dramatically reduce the burden on scientists when it comes to metabolite ID. These metabolite ID inferences could only be made stronger by also integrating knowledge of the retention and elution principles of the chromatographic method applied.

While the application of machine learning to predict retention times in metabolomics is not widespread, published models show promise in ranking putative identities of MS features [22]. These methods aim to establish relationships between calculated molecular descriptors (MDs) and retention time using data sets ranging from <100 [23] to 904 [24] metabolites, and generally <20 MDs. The addition of MDs based on 3D Molecular Interaction Fields, or Volsurf+, expanded the descriptor space and have been applied to promote the correct molecule for improved identification of MS features [25,26]. A quantum leap beyond those previously published, fingerprints describing 80,038 analytes with documented retention times fed a deep learning model, creating the METLIN SMRT database [27], which has now been extended to nano-LC as well [28].

With the goals of designing and modeling structure-dependent separation to chromatographically resolve much of central carbon metabolism and related metabolites, we designed the Mixed Mode (MM) method coupled to negative mode Orbitrap MS. The method is demonstrated to retain 398 of the ~600 unique metabolites of the MSMLS^TM^ library. We then went on to split the retention time (RT) dataset into training and test sets and trained a message-passing neural network (MPNN) model to predict RT from featurized heavy atom connectivity graphs. Comparing predicted to measured RTs, the model showed <2 min RT predictions for a significant proportion of the test set. The model was then applied to determine the most probable metabolite IDs for MS features altered by GLS2 KO.

## 2. Results

### 2.1. Mixed Mode Chromatography for Central Carbon Metabolite Detection

**Figure 1:**
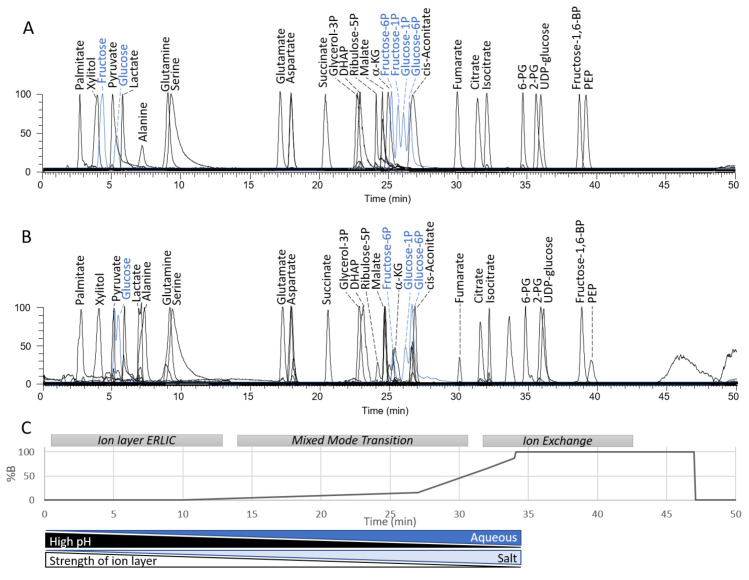
Mixed mode chromatographic resolution of central carbon metabolites (**A**) Extracted Ion Chromatograms (EICs) of standards from central carbon metabolism. (**B**) EICs of the same metabolites from mouse heart. (**C**) Schematic showing MM chromatographic segments and gradient. Applied to a positively charged quaternary amine polyvinyl alcohol stationary phase, Buffer A consists of 90% acetonitrile and 10% water, containing 20 mM Triethylamine: Formic acid at pH 9.18. Buffer B consists of 5% acetonitrile and 95% water containing 54 mM Triethylamine: Formic acid at pH 3.03. Abbreviations: P phosphate; BP bisphosphate, DHAP dihydroxyacetone phosphate; αKG α-ketoglutarate or 2-oxoglutarate; 2PG 2-phosphoglycerate; PEP phosphoenolpyruvate, UDP uridine diphosphate.

Designed to retain the metabolites of central carbon metabolism, which include simple sugars, sugar phosphates, carboxylic acids, and related amino acids, this Mixed Mode chromatography applies varied pH, salt concentration, and organic content to a positively charged quaternary amine polyvinyl alcohol stationary phase (Figure 1).

In the initial ion layer-ERLIC chromatographic segment, under high pH (9.18) and high organic (90% acetonitrile), 20 mM triethylammonium-formate (TEA-formate) is introduced to form an ion layer on a positively charged quaternary amine polyvinyl alcohol stationary phase [8]. During this early segment, neutral and weakly anionic metabolites such as glucose, pyruvate, and palmitate are eluted. Glucose and fructose are baseline resolved (Figure 1A, blue).

To induce the mixed mode transition, a gradient is applied, increasing the aqueous and TEA-formate concentrations while reducing the pH. The combination of increasing the mobile phase hydrophilicity and the concentration of the IP reagent (TEA-formate), while also protonating acidic centers enables the stepwise elution of sugar phosphates and dicarboxylic acids. It is also noted that increasing the CH_2_O content leads to stronger retention as exemplified by C_4_ to C_7_ ketoses (Supplementary Figure S1), as would be expected from HILIC chromatography [29]. Fructose and glucose 1- and 6-monophosphates are also well resolved (Figure 1A, blue).

The final segment employs a predominantly aqueous mobile phase with 54 mM TEA-formate, with the pH 3.03, to achieve more classical protonation-dependent anion exchange-based separation. With a pH below the pKa of formate, the ion layer is removed, enabling analytes to directly interact with the cationic stationary phase. During this last segment, strong acids such as isocitrate, fructose 1,6-bisphosphate and PEP are eluted.

As shown in Figure 1A,B, similar chromatographic peak shapes are observed from standards as compared to the biological matrix, which in this case is mouse heart extract.

### 2.2. Method Validation Using Purified Metabolites

Coupling MM chromatography to the Orbitrap QExactive-Plus, linearity, sensitivity, reproducibility, and mass accuracy were tested using a mixture of 33 metabolite standards (Table 1). Replicates of a standard curve ranging from 5.08 nM to 600 μM in 3-fold increments were injected (5 μL) across four different days. Data from all 13 concentrations and blanks were investigated.

Linearity was demonstrated with R^2^ > 0.98 for 30/33, and R^2^ > 0.96 for all 33 standards across on average 3 orders of magnitude (Table 1). Some metabolites displayed two distinct linear ranges, with low and high concentrations best fit separately. For certain metabolites commonly encountered at high concentrations such as lactate or glucose, concentration-dependent alterations in response must be considered with this as with any LCMS method.

Sensitivity was assessed for each metabolite by determining the lower limit of detection (LLOD), or concentration of analyte reproducibly detected above noise (3:1 S:N). Twenty-six of the standards tested demonstrate LOD <1 pmole.

In addition to the performance in various biological sample matrices, the reproducibility of MM chromatography was evaluated by calculating coefficient of variance (CV) of analytes at 18.5 pmole, with the exception of glyceraldehyde 3-phosphate, which was evaluated at 55.5 pmole. With injections spanning across two weeks, standards show a median CV of 12%. Mass accuracy using external calibration was evaluated, and all metabolites tested demonstrate mass error < 3 ppm with a median of 0.49 ppm.

### 2.3. Structural Diversity Assessment

To test the structural diversity potentially captured by the MM method, and to create a database on which a structure predictor model may be built, the MSMLS ™ library (IROA Technologies) was injected at 2.08 μg/mL (5 µL injection volume). This commercially available metabolite library contains >600 unique metabolites classified in 5 major structural and biological categories: (1) carboxylic acids/amino acids, (2) biogenic amines/polyamines, (3) nucleotides/coenzymes and vitamins, (4) mono- and disaccharides, (5) fatty acids/lipids/steroids and hormones.

Among the 607 unique metabolites tested, 398 are chromatographically well retained, providing 65.6% coverage of the total library. A complete list of retention times is included as Supplemental Table S1. Of those metabolites not detected, 8 fall outside of the *m*/*z* scan range (65–975 *m*/*z*), and the remaining 209 are either not well retained chromatographically or not observed. Metabolites not amenable to this MM method include those which are chemically unstable across the pH gradient (i.e., ATP, GTP, etc.), and those not amenable to ESI negative mode detection (i.e., squalene, choline, most monoamines, sterols, etc.).

### 2.4. Mathematical Model for Structure Digitization and Prediction

We next strove to develop a mathematical model capable of expanding to all 398 diverse metabolites detected from the MSMLS library. Such a model may then be applied to predict structures corresponding to mass spectral features of unknown identity, generating a powerful tool for untargeted/shotgun metabolomics.

**Figure 2:**
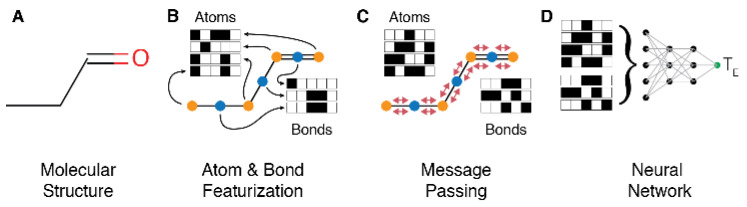
Model for predicting elution times from molecular structures: (**A**) The 2D representation of the molecule is used as input and converted into a connectivity graph. (**B**) Atom and bond properties are extracted from the connectivity graph. (**C**) Properties from neighboring atoms and bonds are transmitted and mixed via four rounds of message passing. (**D**) The final properties at each atom and node are collated and passed through a four-stage set2set neural network to predict the MM retention time (TE).

We trained a message passing neural network (MPNN) [30] to predict the MM retention time given graph representations of molecular structures (Figure 2). RDKit v2017.09 was used to generate heavy atom connectivity graphs from SMILES strings. This connectivity graph was used to construct bond features (bond type, conjugation, presence in ring) and atom features (atomic number, degree, valence, formal charge, number of radical electrons, hybridization, and aromaticity) for every heavy atom and bond between heavy atoms in the molecule. A MPNN was constructed using the DeepChem v2.0.0 library, then trained to infer the MM retention time from this featurized connectivity graph using four rounds of message passing. During each stage of message passing, information from each node (atom or bond) is transmitted to and aggregated at all the neighboring nodes that are one hop away. This process is responsible for the MPNN’s ability to ‘learn’ localized chemical environments throughout a molecule. The message-passing phase was followed by a 4-stage set2set readout during which the information aggregated at each bond and atom is consolidated in a permutation-invariant fashion and used to predict a retention time [31]. This final phase is critical to the MPNN’s ability to accommodate graph isomorphism.

#### 2.4.1. Model Training

We split the dataset of 398 points into training and test datasets in the ratio 318:80 (≈80%:20%). The model was trained on the training dataset for 100 epochs with a batch size of 64. The trained model was then evaluated on predictive accuracy both on the training set and the held-out test set. To estimate the robustness of the model’s predictive accuracy to the choice of training set, we carried out a five-fold cross validation study using five different randomly generated training and test sets. The total training time for a single MPNN model on an NVIDIA K80 GPU was approximately 30 min.

#### 2.4.2. Model Performance Predicting RT

**Figure 3:**
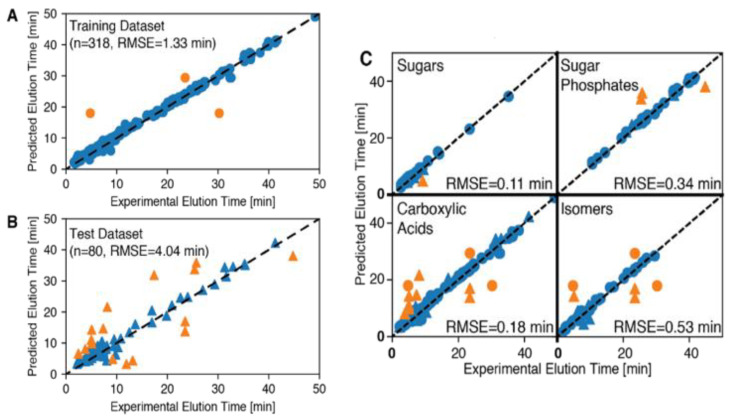
Model performance: (**A**) The performance of the trained MPNN on the training dataset (with 318 data points). Blue points represent data for which the model error is <2 min and orange points represent data where the model error ≥2 min. The dashed black line represents line of zero error. The model RMSE on the training dataset is 1.33 min. (**B**) The performance of the trained MPNN on the held-out test dataset (with 80 data points). The RMSE on this test dataset is 4.04 min. (**C**) The model performance on four different sets of compounds: sugars, sugar phosphates, carboxylic acids, and isomers. Circles represent points that the MPNN model was trained on. Triangles represent points from the held-out test dataset.

Figure 3 shows the results of fitting an MPNN model to the training set and its predictions on a held-out test set. The model root mean square errors (RMSEs) on the training and test datasets are 1.33 min and 4.04 min, respectively. Five-fold cross-validation tests (Supplemental Table S2) indicate that the estimated RMSE on held-out test datasets for the MPNN model (4.62 ± 0.7 min) is superior to the linear regression and random forest models with hand-picked features. Additionally, on average, MPNN models achieve errors of <2 min on a significantly higher fraction (91.2%) of the complete dataset as compared to conventional linear regression and random forests.

Table 2 presents the performance of the MPNN model on selected subsets of the metabolites detected from the MSMLS library (sugars, sugar phosphates, carboxylic acids, and isomers). The model achieves an error of <2 min on 91%, 50%, 77%, and 72% of held-out compounds from these subsets, respectively, with overall RMSEs of 0.11, 0.34, 0.18, and 0.53 min respectively.

#### 2.4.3. Identification of Important Functional Groups

It is possible to interrogate the MPNN model to determine what it has ‘learnt’ with respect to the relationship between molecular structure and retention time. To obtain the model’s estimate of the effect *є_i_* of a given atom *i* on the retention time *E_T_* of a molecule, we set the feature vector corresponding to that atom to a vector of zeros during the featurization stage (Figure 2B) of the inference process. In effect, this replaces the chosen atom in that molecule with a ‘ghost’ atom that has no properties of its own. We then run this molecule with the ghost atom through our model and compute a new retention time $E_{T}^{i}$. The change in the model’s predicted retention time is then attributed entirely to the effect of ‘removing’ the chosen atom, and the difference ${є}_{i}=E_{T}-E_{T}^{i}$ is determined to be the effect of atom *i* on the retention time of that molecule.

We then examined the functional groups that the MPNN identified as the most significant determinants of retention time. From the connectivity graph of each molecule, we computed all unique subgraphs (ignoring ring fragments) with up to five heavy atoms. We then assigned $\sum {є}_{i}$ (where the sum is over all constituents of the subgraph) as the total effect of each subgraph. We then identified the functional groups (identified as chemically unique and sensible subgraphs) with the most positive and most negative total effect on retention time. Supplemental Table S3 in the Supporting Information reports the total effects on retention time for all subgraphs that appear at least 30 times over our entire dataset of 398 molecules and functional groups have been highlighted in bold.

**Figure 4:**
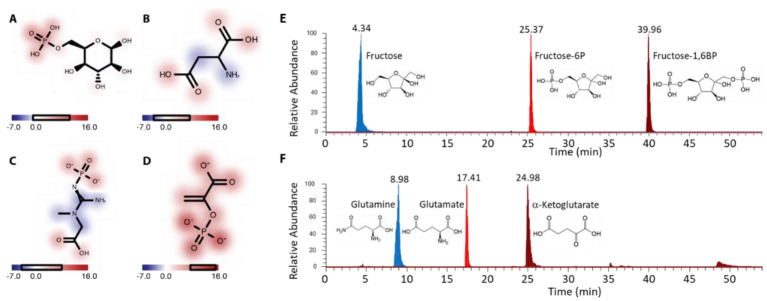
The effects of different functional groups on retention time: In each subfigure, atoms are colored by their effect *є* on the MM retention time, with atoms colored red increasing the retention time and atoms colored blue decreasing the retention time. The color bar indicates the range of *є* in minutes. Phosphate groups are readily identified in (**A**,**C**,**D**) as strongly increasing retention time. Carboxylic acids contribute to a lesser degree as seen in (**B**–**D**) while amine and amide groups are observed to strongly contribute to decreasing elution time in (**B**,**C**). Empirically, (**E**) phosphate increases retention of fructose to F6P and FBP, whereas (**F**) replacing a ketone or carboxylic acid with an amine or amide, respectively, reduces retention from aKG to Glu to Gln.

According to the MPNN model, the functional groups that contribute the most to reducing the retention time are amides and amines, with amides reducing the retention time by approximately 7 min. On the other hand, the MPNN identifies phosphate groups and carboxylic acids as having the greatest effect on increasing the retention time with effects of approximately 15 min and 4 min, respectively. Figure 4A–D depicts the effects of various atoms on the retention time in four representative molecules that contain multiple of these functional groups. Experimental data showing enhanced retention upon phosphorylation of fructose (Figure 4E), and reduced retention by replacing a ketone with an amine and a carboxylic acid with an amide (Figure 4F and Figure S2) further exemplify these points.

### 2.5. Application to GLS2 KO Mouse Primary Hepatocytes

**Figure 5:**
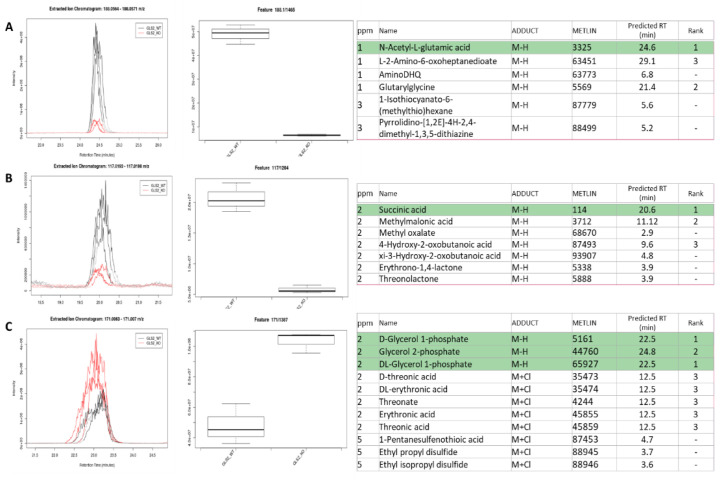
Application of the MPNN model to predict the identities of features altered by GLS2 KO in primary mouse hepatocytes, *p* < 0.01, fold change >2, signal intensity >1E6. (**A**–**C**) represent the most significantly altered features’ extracted ion chromatograms (EICs), box plots, and putative identities as deduced using XCMS.

To assess model performance with biological data, we isolated hepatocytes from GLS2 knockout and wild-type mice, and briefly applied media lacking l-glutamine. Sixty minutes after resupplying Gln, metabolites were extracted and analyzed with the MM method. Data were processed through XCMS, and features were filtered for *p* < 0.01, fold change >2, and a minimum intensity of 1 × 10^6^. Sorting by smallest *p* value, the first extracted ion chromatogram (EIC) with good chromatographic peak shape corresponded to 188.0567 *m*/*z* at 24.41 min. Six putative IDs were within 3 ppm of the experimentally observed *m*/*z*, representing two chemical formulas (Figure 5A), none of which had documented retention times in training or test sets. Amongst these potential IDs, the model correctly predicted n-acetyl-l-glutamic acid as the most likely candidate, as verified by injection of purchased standards.

The next most significant difference between GLS2KO vs. WT was 117.0196 *m*/*z* observed at 20.07 min. The model had been trained on 2/6 of the putative IDs. Despite the four additional isomers suggested, the model correctly selected succinate as reduced by GLS2 KO (Figure 5B).

The third most significant hit corresponds to 171.0068 *m*/*z* at 23.11 min. Although glycerol 1-p and 2-p are both potential hits, almost indistinguishable by the model, the large gap in retention times between these top hits and the Cl- adducts of threonate (and isomers) is apparent, further supporting the correct identification as glycerol monophosphate (Figure 5C). Expansion of the list to include *p* < 0.05 leads to the identification of Glutamine, Glutamate, and other downstream metabolites known to be altered by GLS2 KO. The complete results table generated from XCMS is included as Supplemental Table S4.

## 3. Discussion

Given how dramatically advancements in mass spectrometry have enabled enhanced selectivity and specificity in the field of metabolomics, we believe that continual chromatographic improvement coupled to MPNN modeling offers the opportunity for a parallel advancement in our ability to differentiate and predict the identities of MS features. Although not able to capture the totality of the defined metabolome, as no current method can, the breadth of coverage and ability to separate isomers makes the MM method reported herein an important contribution towards the evolution of comprehensive metabolomics methods.

One advantage of this MM method is the ability to differentiate isomers, which is truly critical to understand regulation and dysregulation of central carbon metabolism. For instance, while glucose is converted to glucose 6-phosphate and proceeds through glycolysis in a tightly controlled fashion, its isomer fructose is converted to fructose 1-phosphate by ketohexokinase (KHK), which lacks any negative feedback control. This occurs rapidly even with low amounts of fructose, resulting in the depletion of ATP, fructose-induced nucleotide turnover, and uric acid production, contributing uniquely to the pathogenesis of diabetes and obesity [32]. Additionally, excess sugar intake in the form of sugar sweetened beverages has been linked to a multitude of diseases including T2D [33,34], CVD [35], and all-cause mortality [36,37]. Contrast each of these catabolic routes with the branching of glucose via glucose 1P into glycogen, a major energy storage polymer in muscle and liver. While studying concentrations and fluxes through these central metabolic pathways, failure to separate hexoses and hexose monophosphates from one another leads to dilution signal from coeluting isomers, where the highest concentration hexose (monophosphate) dominates and obscures changes in others.

Although the MM retention times of >400 metabolites are reported here, one notable metabolite of importance that is not captured by the method is ATP. The model predicts a RT of 41.2 min, indicating that it should elute earlier than the six other metabolites in the training set. It is likely that the pH gradient applied leads to degradation of ATP, which may also artificially inflate ADP and AMP measurements. Additionally, for chemically labile metabolites, a 60 min gradient is less than ideal. For these reasons we recommend running a separate method for determination of energy charge ([ATP] + 0.5 * [ADP]/[AMP]).

Having trained and tested the MPNN model on 398 metabolite retention times, it is noted that not all structural features of the metabolome are represented in the training and test sets. One example of an analyte not included in the MSMLS library but serving a central role in the urea cycle is l-arginosuccinate. While both arginine and succinate are represented in the training set, the N-linkage of the guanidino group to a dicarboxylic acid is not. Two features at 20.1 and 28.7 min were observed in the mouse hepatocyte extracts reported herein, both of which match the exact mass of arginosuccinate. The model predicts a retention time of 21.9 min, more closely matching the former, however upon injection of a pure standard the RT it was found to be ~28 min, indicating that the latter is more likely to represent endogenous l-arginosuccinate. This example demonstrates that additional training of expanded chemical space would further enhance the model’s predictive ability.

We believe that coupling documented RTs to modeling will provide predictive power and enhanced confidence in metabolite ID. Graph-based neural network models have recently been shown to not only achieve state-of-the-art accuracies on predictive cheminformatics benchmarks, but to also be capable of inferring structure-property relationships without needing to be provided with conventional, hand-crafted MDs [30,38]. By operating directly on the chemical graph of molecules, these methods also surpass fingerprint-based models that are hampered by the sparsity and noise inherent to conventional chemical fingerprints [39]. The availability of user-friendly, well-documented implementations of these networks as open-source software libraries (e.g., https://deepchem.io, accessed on 10 November 2021) makes the application of these methods to structure-property relationship modeling as easy as the application of conventional QSAR tools and packages.

The low RMSE values on the complete dataset and good performance on the test datasets indicate that the MPNN can accurately deduce structure-property correlations for almost all of the training dataset and most of the test dataset. Furthermore, the MPNN demonstrably performs better than conventional QSAR methods on this dataset. However, higher errors for some compounds on the test dataset indicate that the MPNN has been unable to learn all of the interactions between functional groups on certain molecules. Indeed, most of the compounds for which the MPNN produces predictions with errors >2 min are characterized by the presence of intra-molecular hydrogen bonds such as maleic acid. Although the MPNN does not perceive the conformational geometry and resultant 3D intramolecular interactions between various moieties (a limitation shared by conventional QSAR methods), we hypothesize that it will become possible to increase the number of message passing steps and potentially learn long-range interactions such as intramolecular hydrogen bonding.

While some of the model’s conclusions, such as increased acidity enhancing retention, will come as no surprise to the experienced analytical chemist, it is important to note that our model was provided with no a priori knowledge of chemistry whatsoever. These estimates of effects on retention time were obtained from a model that was provided with no human-specified chemical features or fingerprints, operating purely on a graph representation of molecular structures. This ability to assign effects on retention time to functional groups in a manner that is consistent with experimental chromatography demonstrates that the MPNN model is not only accurate but interpretable, enabling its potential use in structure determination in untargeted/shotgun metabolomics studies.

## 4. Materials and Methods

### 4.1. Chemicals

Acetonitrile, methanol, and water, all Optima^TM^ LC-MS grade, were purchased from Fischer Scientific (Pittsburgh, PA, USA). LC-MS mobile phase additives, such as 2 M Formic acid: Triethylamine (1:1) solution, formic acid, trimethylamine and standard compounds (Supplemental Methods) were purchased from Sigma-Aldrich (St. Louis, MO, USA). Mass Spectrometry Metabolite Library of Standards (MSMLS^TM^) was purchased from IROA Technology (Sea Cirt, NJ, USA).

### 4.2. Standard Solutions and Sample Preparations

Standard stock solutions were initially prepared in water, then diluted into 35:40:25 acetonitrile:methanol:water for injection. The MSMLS^TM^ standards were prepared following IROA instructions with slight modifications. Ten µL methanol was dispensed into each well of MSMLS 96 well plates (1 to 7). Methanol was allowed to incubate in plates 6 and 7 for 2 h. Then, acetonitrile, methanol and water were added to a final ratio of 35:40:25 acetonitrile:methanol:water (plates 1–5) and 9:8:3 acetonitrile:methanol:water (plates 6–7). Wells were pooled to create 56 injections, avoiding the pooling of isomers.

### 4.3. LCMS

The LC-MS platform consists of a Q Exactive Plus Orbitrap Mass Spectrometer with enhanced MS resolution up to 280,000, coupled with a Dionex UltiMate 3000 RSLC system, including binary pump, column compartment and autosampler (Thermo Fisher Scientific, San Jose, CA, USA). Liquid chromatography separation was achieved on a HILICpak VT50 2D column (150 mm × 2.0 mm, 5 µm particle size, Shodex, Japan). Buffer A consists of 90% acetonitrile, 10% water, containing 20 mM Triethylamine: Formic acid at pH 9.18; Buffer B consists of 5% acetonitrile, 95% water containing 54 mM Triethylamine: Formic acid at pH 3.03. Flow rate is 0.2 mL/min from 0 to 5 min, then 0.3 mL/min from 5.1 to 58 min, and reduced again to 0.2 mL/min from 58.1 to 60 min. The gradient starts with 0%B from 0 to 10 min, then increases linearly from 0 to 16%B from 10 to 27 min, up to 65%B at 32 min, 87%B at 34 min, 100%B hold from 34.1 to 47 min, then 0%B from 47.1 to 60 min.

The Mass Spectrometry parameters are set as Source Fragmentation: None; Sheath gas flow rate: 45; Aux gas flow rate: 15; Sweep gas flow rate: 3; Spray voltage: 3.00 kV; Capillary temp: 310 °C; S-lens RF level: 50; Aux gas heater temp: 350 °C; For Full MS: Scan range: 65.0 to 975.0 *m*/*z*; Resolution: 140,000; Polarity: Negative; AGC target: 3e6; Maximum IT: 500 ms.

Peak identification was conducted by applying Thermo Xcalibur^TM^ Qual browser and MAVEN (version x64_774) [40,41]. For differential analysis of GLS2KO vs. WT, mzxml files were uploaded to XCMS for pairwise analysis. Parameters assigned to the method include feature detection with centWave, applying 2.5 ppm maximum tolerated *m*/*z* deviation in consecutive scans, peak with between 10 and 60 s, and prefilter intensity ≥5000 with orbiwarp RT correction, step size 1 *m*/*z*. An alignment mzwidth of 0.015 and 5 s allowable RT deviations were applied. Annotation and identification were limited to isotopes and adducts within 5 ppm tolerance for the database search. GLS2 WT vs. KO data is also available at the NIH Common Fund’s National Metabolomics Data Repository (NMDR) website, the Metabolomics Workbench, https://www.metabolomicsworkbench.org (accessed on 10 November 2021) where it has been assigned Project ID PR001239. The data can be accessed directly via its Project DOI: http://dx.doi.org/10.21228/M8JT51 (accessed on 10 November 2021). This work is supported by NIH grant U2C-DK119886.

### 4.4. Hepatocyte and Tissue Isolation and Treatment

Primary hepatocytes were isolated from male mice between 12 and 18 weeks of age by the two-step collagenase perfusion method. Mice were fasted 16 h before the experiments. After isolation, cells were plated in M199 media with 10% FBS for 4 h in 6-well plates pre-coated with collagen I. After cells were attached to the plates, they were washed with glucose output media (GOM) (118 mM NaCl, 4.7 mM KCl, 1.2 mM MgSO_4_, 1.2 mM KH_2_PO_4_, 1.2 mM CaCl_2_, 20 mM NaCO_3_, 25 mM HEPES pH 7.4, and 0.025% BSA), and incubated in fresh GOM for 2 h. GOM media was replaced with fresh, pre-warmed GOM media and cellular treatments initiated. Hepatocytes were treated with 5 mM unlabeled glutamine for 60 min. Hepatocytes were washed with ice cold PBS twice and immediately frozen in liquid nitrogen. Mouse heart was flash frozen upon resection.

### 4.5. Cell and Tissue Extraction

Flash frozen mouse heart tissue or hepatocytes were extracted on dry ice with 80:20 methanol:water, vortexed, centrifuged at 14,000× *g* at 4 °C for 15 min, dried under nitrogen gas, and reconstitution in 35:40:25 acetonitrile:methanol:water for injection.

### 4.6. Message Passing Neural Network (MPNN)

MPNNs are defined on a graph (containing nodes and edges) by (i) an aggregation function that aggregates features from neighboring nodes and edges, (ii) an update function that updates the features of a node using the aggregated features of neighboring nodes and edges, and (iii) a readout function that combines the features of all nodes on the graph to generate a final feature vector that represents the entire graph. MPNNs have demonstrated state-of-the-art accuracies on regression tasks on datasets of even just a few hundred compounds [42]. The DeepChem Python library contains an easy-to-use implementation of MPNNs (among other neural network architectures) modified to operate on molecular graphs. The MPNN in this work used Edge Networks as the aggregation function, a Gated Recurrent Unit for the updates, and concatenation followed by input through a single perceptron with ReLu activation as the readout function (i.e., DeepChem defaults). Additional details may be found on the DeepChem MPNNModel documentation [43]. Scripts for training and evaluating the MPNNs and generating the figures in this manuscript may be found at https://github.com/PfizerRD/mixed-mode-mpnn (accessed on 10 November 2021).

## 5. Conclusions

Despite decades of significant methodological progress in the field of metabolomics, continued evolution to improve the selectivity and specificity, while also capturing an even broader chemical space, is paramount. Combining new methods with the ability to make informed predictions in the identification of ‘unknowns’ holds great promise for expanding and automating metabolite ID in the future. Although limited to negative mode, this MM method enables the unambiguous identification of >400 metabolites. Combined with graph-based neural network models using featurized connectivity graphs, this method provides the foundation for improved LC predictive power. Expansion of the MPNN-enabled, structure-based modeling will be a topic of future publication as we strive to expand the training set and apply to additional analytical methods.

## Figures and Tables

**Table 1:** Method sensitivity and reproducibility.

Metabolite	LOD (pmole)	CV (n = 4)	Linear Range (pmole)	Linear Coefficient (R^2^)	Mass Error (ppm)
2-phosphoglycerate	0.0254	10.8	0.229–1000	0.98729	0.54
6-phosphogluconate	0.076	10.7	0.229–500	0.9993	0
a-ketoglutarate	0.685	15.8	2.06–1500	0.99622	0.69
L-alanine	6.15	20.9	6.15–1500	0.99199	2.27
L-aspartate	0.076	9.15	0.229–1500	0.99862	0
cis-aconitate	0.0254	39.3	0.0760–166.5	0.99015	0.58
DHAP	18.5	33.2	18.5–1500	0.99815	0.59
sedoheptulose 7P	0.076	12.8	0.229–500	0.9981	0.69
fructose-13C6	0.0254	12	0.0254–166.5	0.99749	0.54
fructose-1,6-BP	0.0254	17.6	0.0254–1500	0.99259	0.59
fructose-1P	0.0254	9.62	0.0760–1500	0.99408	0
fructose-6P	0.0254	12.8	0.0760–1500	0.99078	0
fumarate	0.685	11.3	2.06–1500	0.98993	0
glucose	0.685	13.8	2.06–1500	0.98751	0.56
glucose-1P	0.0254	12.3	0.0760–500	0.9955	0
glucose-6P	0.0254	11.4	0.0760–500	0.99585	0
glutamate	0.0254	8.95	0.229–3000	0.99572	−0.68
glutamine	0.076	6.85	0.229–1500	0.99001	−0.69
glyceraldehyde-3P	55.5	5.45	55.5–1500	0.98939	0.59
glycine	18.5	4.71	18.5–1500	0.99478	0
glyecrol-3P	0.076	7.95	0.229–500	0.99723	0.58
isocitrate	6.15	31.6	6.15–500	0.96498	0.52
lactate	0.229	z12.4	0.229–55.5	0.98791	0
malate	0.076	39.2	0.229–166.5	0.99544	0.75
myo-inositol-d6	6.15	15.1	6.15–1500	0.9838	0.54
phosphoenolpyruvate	0.076	14.3	0.229–1500	0.99564	0.6
pyruvate	2.06	18.9	2.06–1000	0.96721	0
ribulose-5P	0.685	9.84	0.685–1000	0.98069	−0.44
serine	0.229	9.88	0.229–1500	0.99155	0.96
sorbitol	0.076	11.2	0.229–500	0.99293	0
succinate	0.229	12.2	2.06–1000	0.96498	0
UDP-glucose	0.229	12.1	0.229–1500	0.99135	1.77
xylitol-13C5	0.229	9.35	2.06–1000	0.98754	0.64

**Table 2:** Model performance on selected subsets of metabolites detected from the MSMLS library.

	Training		Test	
Subset	Dataset Size	# Points with Error <2 min	Dataset Size	# Points with Error <2 min
Sugars	55	55	11	10
Sugar-P	39	39	6	3
Carboxylic acids	148	145	35	27
Isomers	37	34	11	8

## Data Availability

GLS2 WT vs KO data is also available at the NIH Common Fund’s National Metabolomics Data Repository (NMDR) website, the Metabolomics Workbench, https://www.metabolomicsworkbench.org (accessed on 10 November 2021) where it has been assigned Project ID PR001239. The data can be accessed directly via it’s Project DOI: http://dx.doi.org/10.21228/M8JT51 (accessed on 10 November 2021). This work is supported by NIH grant U2C-DK119886. Scripts for training and evaluating the MPNNs and generating the figures in this manuscript may be found at https://github.com/PfizerRD/mixed-mode-mpnn (accessed on 21 October 2021).

## Supplementary Materials

The following are available online at https://www.mdpi.com/article/10.3390/metabo11110772/s1, Figure S1: Elution order of C4 to C7 monophosphorylated ketoses, Figure S2: Replacing -COOH with an amide leads to earlier elution of Asn relative to Asp, Table S1: Retention times of MSMLS library, Table S2: Performance of assorted ML methods on the elution data set. Table S3: The effect ∑*є_i_* of various subgraphs. Table S4: XCMS results table output. Methods S1: Mobile phase preparation.
