# Supplementary material for: Coupling Mixed Mode Chromatography/ESI Negative MS Detection with Message-Passing Neural Network Modeling for Enhanced Metabolome Coverage and Structural Identification
Source: Metabolites. 2021 Nov 11;11(11):772. doi: 10.3390/metabo11110772 (PMC8620857; doi:10.3390/metabo11110772)
Supplement: Supplementary file 1 [file metabolites-11-00772-s001.zip › Xing et al Supplemental figures.pdf]

Supplemental Figures:

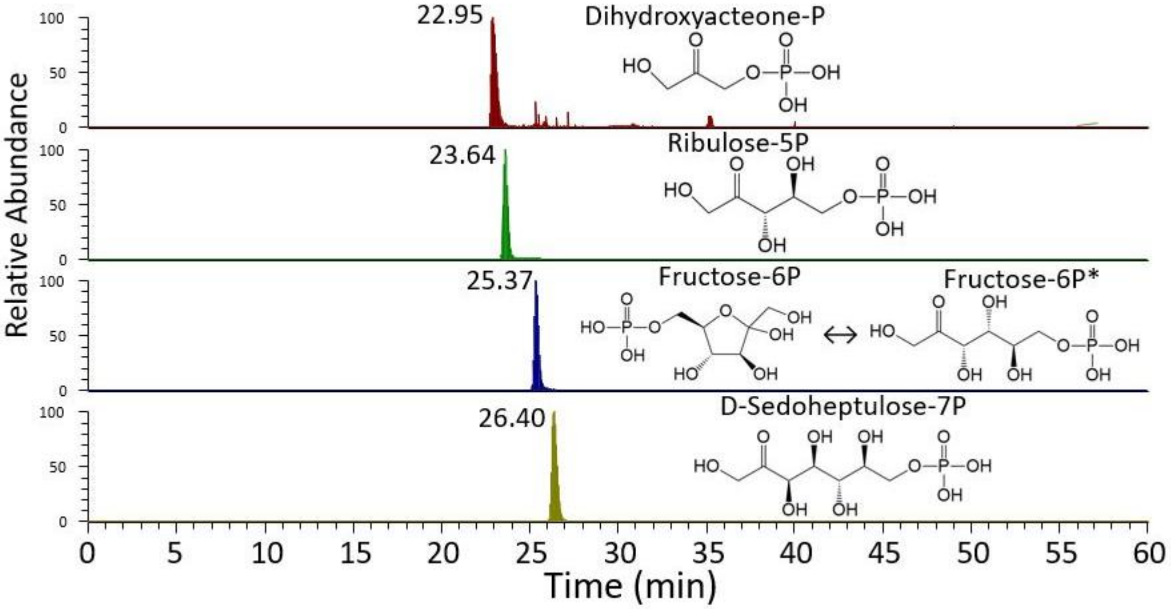

Figure S1. Elution order of C4 to C7 monophosphorylated ketoses: More CH_2O content leads to stronger retention: C3 (dihydroxyacetone phosphate, DHAP) < C5 (xylulose-5P/ribulose-5P) < C6 (fructose-6P/fructose-1P) < C7 (sedoheptulose-7P).

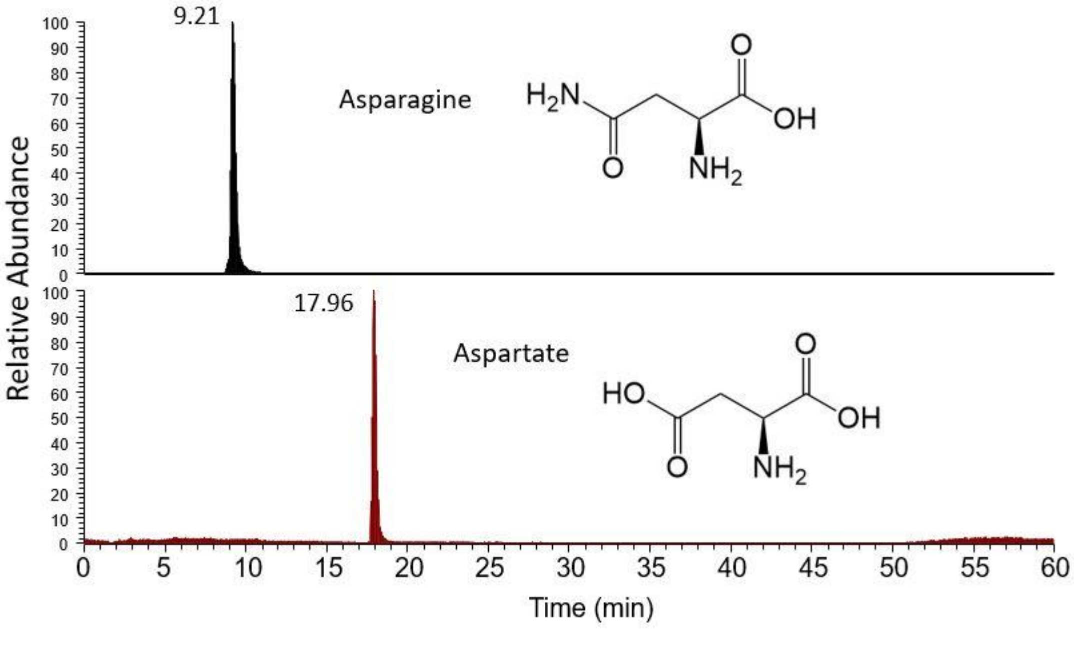

Figure S2. Replacing -COOH with an amide leads to earlier elution of Asn relative to Asp.
