# Supplementary material for: Coupling Mixed Mode Chromatography/ESI Negative MS Detection with Message-Passing Neural Network Modeling for Enhanced Metabolome Coverage and Structural Identification
Source: Metabolites. 2021 Nov 11;11(11):772. doi: 10.3390/metabo11110772 (PMC8620857; doi:10.3390/metabo11110772)
Supplement: Supplementary file 1 [file metabolites-11-00772-s001.zip › Xing et al Supplemental methods.pdf]

**<u>Methods S1:</u>**

**Mobile phase preparation**

To make a 100 mL 2 M Triethylamine-formic acid stock, 28.02 mL Triethylamine (Supelco 81101) was added into a clean graduated bottle on ice with stirring, first followed by slow addition of 7.70 mL formic acid (Supelco 53302), then 60 mL HPLC grade water (Optima @ LC/MS W6-4 from Fisher). The pH of resulting solution was adjusted to 6.54 using formic acid, prior to being transferred into a clean graduated cylinder and filled up to 100 mL by HPLC grade water.

*Preparation of buffer A (90% acetonitrile, 10% water, 20 mM Triethylamine-Formic acid at pH 9.18)*

900 mL HPLC grade acetonitrile (Optima @ LC/MS A955-4 from Fisher) was added into a clean graduated cylinder, followed by 50 mL HPLC grade water, then 10 mL of 2 M Trietheylamine : Formic acid stock. The solution was then transferred into a clean 1 L beaker. Under stirring, the solution pH was adjusted to 9.18 with Triethylamine prior to being transferred back to the graduated cylinder and filled up to 1 L by HPLC grade water.

*Preparation of buffer B (5% acetontrile, 95% water, 54 mM Triethylamine-Formic acid at pH 3.03)*

50 mL HPLC grade acetonitrile was added into a clean 1 L graduated cylinder, followed first by HPLC grade water up to 900 mL measure line, then 27 mL 2 M Triethylamine-Formic acid stock. The solution was transferred into a clean 1 L beaker, and under stirring, pH was adjusted to 3.03 using Formic acid. The final volume was adjusted to 1 L in the graduated cylinder by filling up HPLC grade water.
