# Supplementary material for: Coupling Mixed Mode Chromatography/ESI Negative MS Detection with Message-Passing Neural Network Modeling for Enhanced Metabolome Coverage and Structural Identification
Source: Metabolites. 2021 Nov 11;11(11):772. doi: 10.3390/metabo11110772 (PMC8620857; doi:10.3390/metabo11110772)
Supplement: Supplementary file 1 [file metabolites-11-00772-s001.zip › Xing et al Supplemental Tables.pdf]

Table S1, see excel file

Table S2: Performance of assorted machine learning methods on the elution data set in 5-fold cross validation. The MPNN performs better than Linear Regression and Random Forest (applied to hand-selected features) on both measures: RMSE and the % of overall predictions with an error of less than 2 minutes.

<table>
<tr><th>Method</th><th>Features</th><th>Mean (STD) RMSE on test dataset</th><th>Mean (STD) % of training + test predictions with error ≤ 2 minutes</th></tr>
<tr><td>Linear Regression</td><td rowspan="2">Number of basic centers, number of acidic centers, radius of gyration, total polar surface area, total atom information content[1], total heavy atom bonds</td><td>4.68 (3.15)</td><td>78.8</td></tr>
<tr><td>Random Forest</td><td>4.88 (2.72)</td><td>85.4</td></tr>
<tr><td>MPNN</td><td>Weave Features</td><td>4.62 (0.7)</td><td>91.2</td></tr>
</table>

Table S3: The effect $\sum \epsilon_i$ of various subgraphs (with fewer than 6 atoms) to the elution time. Only subgraphs that occur more than 30 times in the entire dataset are included. Aromatic ring fragments are excluded.

Subgraph	Mean Total Effect (min)	Mean Absolute Efect (min)	Mean Relative Effect	Mean Absolute Relative Effect	Frequency	Annotation
**C=CCCC**	-7.42	9.52	-2.83	2.98	33	
**CC=CCC**	-5.96	8.10	-2.37	2.52	48	
**CC(C)CO**	-9.96	11.31	-2.28	2.34	37	
**cccC=O**	-7.01	12.72	-1.95	2.31	52	
**CCNCC**	-9.39	10.83	-1.88	1.95	61	
**cccCO**	-4.08	14.45	-1.75	2.34	46	
**C=CCC**	-4.89	6.00	-1.73	1.81	45	
**C1CCOC1**	-11.15	12.07	-1.68	1.71	54	

[1] As defined by a_IC at https://www.chemcomp.com/journal/descr.htm

**C(Cn)CO**	-9.88	11.12	-1.62	1.65	50	
**CCCNC**	-7.72	9.81	-1.60	1.68	46	
**CC(=O)NC**	**-6.81**	**9.99**	**-1.57**	**1.71**	**32**	**Amide**
**CCC(n)O**	-10.12	11.35	-1.56	1.59	49	
**CC(n)OC**	-9.97	11.18	-1.55	1.58	49	
**CC(=O)N**	-6.69	9.02	-1.54	1.64	34	
**CC(Cn)O**	-9.41	10.64	-1.54	1.57	38	
**CCOCn**	-9.89	11.05	-1.53	1.56	98	
**CC(nc)O**	-9.56	11.12	-1.52	1.56	98	
**COCnc**	-9.35	10.91	-1.50	1.54	98	
**CC(C)NC**	-8.43	8.89	-1.49	1.52	33	
**cnCCO**	-8.82	10.35	-1.48	1.52	76	
**cn(c)CO**	-8.93	10.81	-1.47	1.52	49	
**ccCO**	-3.46	11.71	-1.42	1.91	52	
**C(ncn)O**	-7.23	9.86	-1.42	1.48	74	
**CNCC=O**	-6.39	8.94	-1.41	1.54	44	
**CCNC**	-7.28	8.47	-1.37	1.43	124	
**ccnCO**	-8.27	10.33	-1.36	1.41	52	
**ccC=O**	-4.63	9.31	-1.32	1.63	59	
**C(C(n)O)O**	-7.39	8.87	-1.29	1.32	37	
**CC(n)O**	-7.81	8.94	-1.25	1.27	49	
**CCC(C)O**	-6.88	8.15	-1.24	1.29	691	
**CCNC=O**	-5.92	7.94	-1.22	1.32	68	
**COCn**	-7.61	8.73	-1.22	1.25	49	
**CCCC=O**	-3.27	8.72	-1.22	1.45	211	
**CC(C=O)O**	-1.65	10.77	-1.20	1.51	47	
**cnCO**	-7.19	8.68	-1.20	1.23	98	
**C(CO)n**	-6.87	7.97	-1.18	1.21	38	
**CC(C)OC**	-7.68	8.70	-1.18	1.21	101	
**CCOCC**	-7.56	8.72	-1.18	1.21	211	
**ccCCN**	-2.08	14.20	-1.17	1.72	32	
**CNC**	**-6.12**	**7.06**	**-1.09**	**1.14**	**58**	**Secondary Amine**
**C(=O)CO**	-0.86	10.57	-1.08	1.40	66	
**CCCOC**	-6.51	7.72	-1.05	1.09	170	
**CCCCN**	-4.23	10.43	-1.03	1.33	57	
**COCCO**	-5.83	7.19	-0.99	1.04	294	
**CNC=O**	-4.49	6.82	-0.98	1.09	43	
**CCCCO**	-5.15	7.45	-0.97	1.07	803	
**CCOC**	-6.08	7.03	-0.96	0.99	322	
**C(n)O**	-5.45	6.53	-0.92	0.95	49	
**CC(CO)O**	-5.04	6.86	-0.92	0.98	850	

**C(CO)CO**	-4.81	6.74	-0.90	0.97	462	
**CC(C)O**	-5.14	6.30	-0.89	0.94	461	
**CCCN**	-4.29	8.03	-0.88	1.06	101	
**ccC(=O)O**	0.69	10.53	-0.84	1.61	34	
**CCC=O**	-2.92	7.48	-0.83	1.03	250	
**C(=O)N**	-3.43	5.18	-0.81	0.89	51	
**COC**	-4.60	5.35	-0.81	0.83	113	
**CCCO**	-4.45	6.25	-0.80	0.88	1134	
**C=CC**	**-2.50**	**5.58**	**-0.80**	**0.96**	**52**	**Alkene**
**cC=O**	-1.87	6.41	-0.80	1.08	33	
**CCC(C)N**	-4.66	7.96	-0.77	0.95	62	
**C(C(O)O)O**	-3.08	5.20	-0.72	0.81	46	
**CC(C=O)N**	-3.45	8.10	-0.72	0.95	83	
**C(CO)O**	-3.37	5.44	-0.69	0.77	566	
**CC(C)N**	-4.10	7.48	-0.68	0.85	91	
**CC=O**	-1.65	6.22	-0.67	0.87	316	
**CCC(O)O**	-3.11	5.14	-0.66	0.74	45	
**CC(O)OC**	-3.38	5.01	-0.66	0.72	66	
**CNCCO**	-3.99	7.01	-0.64	0.78	42	
**CCN**	-3.18	6.33	-0.63	0.78	248	
**C(C=O)CO**	-1.81	9.45	-0.61	0.86	56	
**CC(CO)N**	-3.88	7.79	-0.61	0.82	88	
**CCOCO**	-3.29	4.99	-0.59	0.65	105	
**CC(O)O**	-2.55	4.31	-0.57	0.64	49	
**CCO**	-2.85	4.73	-0.57	0.65	1406	
**C(C=O)N**	-1.87	6.37	-0.52	0.75	95	
**COCO**	-2.59	4.07	-0.51	0.57	61	
**CN**	**-2.46**	**4.49**	**-0.50**	**0.60**	**197**	**Primary Amine**
**C(O)O**	**-1.64**	**3.38**	**-0.40**	**0.47**	**46**	
**CCC(=O)O**	1.00	9.18	-0.37	0.82	192	
**C(CO)N**	-1.23	6.46	-0.35	0.62	103	
**CO**	**-1.11**	**3.34**	**-0.34**	**0.45**	**984**	**Alcohol**
**C(C(=O)O)N**	-0.19	7.36	-0.32	0.69	84	
**C=O**	**0.52**	**4.46**	**-0.26**	**0.51**	**324**	**Ketone**
**CC(=O)O**	2.58	8.51	-0.21	0.72	218	
**CCCOP**	-0.48	5.86	-0.14	0.30	67	
**C(C(=O)O)O**	9.19	15.78	-0.09	0.93	34	
**CCOP**	1.56	5.46	-0.05	0.24	76	
**C(=O)O**	**3.76**	**7.51**	**-0.04**	**0.60**	**238**	**Carboxylic Acid**
**C(COP)O**	2.52	6.24	-0.02	0.26	70	
**CCOPO**	4.55	7.74	0.06	0.31	150	

**COP**	3.23	4.48	0.07	0.16	66	
**CCOP=O**	5.36	8.01	0.09	0.31	76	
**P**	3.05	3.09	0.10	0.11	67	
**COPO**	6.26	7.28	0.17	0.26	132	
**OP**	5.96	6.05	0.20	0.21	199	
**OPOP**	7.38	7.38	0.20	0.20	56	
**COP=O**	7.16	7.86	0.21	0.27	66	
**O=P**	7.19	7.23	0.24	0.25	67	
**OPOPO**	8.96	8.96	0.24	0.24	56	
**COP(O)O**	9.28	10.26	0.27	0.36	66	
**O=P(O)OP**	10.29	10.29	0.28	0.28	56	
**O=POPO**	10.29	10.29	0.28	0.28	56	
**OPO**	8.77	8.91	0.29	0.30	197	
**COP(=O)O**	10.18	10.91	0.31	0.37	132	
**O=PO**	10.08	10.17	0.33	0.34	199	
**OP(O)O**	11.49	11.69	0.37	0.39	65	
**O=P(O)O**	12.88	13.03	0.42	0.44	197	
**O=P(O)(O)O**	**15.58**	**15.78**	**0.51**	**0.53**	**65**	**Phosphate**

Table S4, see excel file
